# Analysis of the differential expression of serum miR-21-5p, miR-135-5p, and miR-155-5p by Bifidobacterium triplex viable capsules during the perioperative stage of colorectal cancer

**DOI:** 10.1007/s00384-024-04617-8

**Published:** 2024-04-07

**Authors:** Jing Zhang, Ji Guo, Ruochong He, Ji Li, Bingyi Du, Yi Zhang, Rongliang He, Haixia Cheng

**Affiliations:** 1 Department of General Surgery, Shanxi Bethune Hospital, Xiaodian District, No. 99 of Longcheng Street, Taiyuan, 030032 China; 2 Second Department of General Surgery, Shanxi Provincial Integrated TCM And WM Hospital, Taiyuan, 030013 China; 3 Department of General Surgery, Xiaoyi People’s Hospital of Shanxi Province, Luliang, 032300 China

**Keywords:** Bifidobacterium triplex viable capsules, Colorectal cancer, miR-21-5p, miR-135-5p, miR-155-5p, Perioperative period

## Abstract

**Objective:**

In this study, we investigated the impact of perioperative administration of Bifidobacterium triplex viable capsules on the serum levels of circulating miR-21-5p, miR-135-5p, and miR-155-5p in patients with colorectal cancer (CRC). The purpose of this study is to provide a foundation for future research on the use of Bifidobacterium triplex viable capsules to enhance postoperative recovery in patients with CRC.

**Methods:**

A total of 60 patients with primary CRC admitted to the Department of General Surgery at Shanxi Bethune Hospital between June 2020 and December 2020 were selected and randomly divided into two groups: 20 cases in the control group and 40 cases in the experimental group. The experimental group was administered oral Bifidobacterium triplex viable capsules during the perioperative period, while the control group was administered oral placebo. Before and after the perioperative period, the expression levels of miR-21-5p, miR-135-5p, and miR-155-5p were compared in the serum of both groups of patients. Furthermore, we established the prognostic value of these three miRNAs in CRC patients.

**Results:**

After surgery, the expression levels of miR-21-5p, miR-135-5p, and miR-155-5p decreased in both groups of patients (*P* < 0.05). Significantly greater differences were observed between miR-21-5p and miR-135-5p (*P* < 0.001). Expression levels of serum miR-21-5p (*P* = 0.020) and miR-135-5p (*P* = 0.023) decreased significantly more in the experimental group than in the control group. The levels of the above three miRNAs after surgery did not correlate with 3-year OS (HR = 4.21; 95% CI 0.37–47.48; log-rank *P* = 0.20) or 3-year DFS (HR = 1.57; 95% CI 0.32–7.66; log-rank *P* = 0.55) in two groups.

**Conclusion:**

Radical surgery reduces the levels of serum miR-21-5p, miR-135-5p, and miR-155-5p expression in patients with CRC. The use of Bifidobacterium triplex viable capsules assists in achieving quicker perioperative recovery from radical surgery in CRC patients, and this underlying mechanism may be associated with the regulation of serum miR-21-5p, miR-135-5p, and miR-155-5p expression levels.

## Introduction

Colorectal cancer (CRC) is a prevalent malignant tumor caused by flora disequilibrium [1, 2], with the third highest incidence rate (10%) and the second highest mortality rate (9.4%) globally. It poses a serious threat to the lives and health of people around the world [3]. The primary treatment for patients with CRC is surgical resection of tumor lesions. However, perioperative procedures such as preoperative intestinal preparation [4], antibiotic prophylaxis [5, 6], and radical surgery [7, 8] can damage the intestinal barrier, resulting in intestinal flora disequilibrium. It is important to observe that postoperative intestinal flora dysbiosis is strongly associated with the prognosis and recurrence of CRC [9, 10]. In CRC, the perioperative administration of microecological enteral nutrition interventions, such as *Bifidobacterium* and *Lactobacillus*, is a safe and reliable approach for treating postoperative intestinal flora dysbiosis. These interventions play a role in deactivating oncogenic factors, regulating host immunity, controlling apoptosis and proliferation, and improving the integrity of the intestinal mucosa through multiple mechanisms [11, 12]. However, the specific mechanisms underlying these effects are not yet completely understood [13].

Recent research on the role of probiotics in CRC has gradually expanded from protein-coding genes to non-coding RNAs, such as microRNAs (miRNAs), long intergenic non-coding RNAs, and circular RNAs [1, 2]. miRNAs usually bind to the 3′-untranslated area (3′-UTR) of a specific target mRNA. This binding inhibits messenger RNA (mRNA) translation through degradation of the mRNA and further contributes to the development of cancer. In cancer development, dysregulation of transcription and aberrant expression of miRNAs can have oncogenic or carcinogenic effects, thereby regulating the progression of tumors. Primarily, these effects involve mechanisms such as regulating cell proliferation, inhibiting cell death, controlling tumor invasion and metastasis, and inducing angiogenesis [14].

Recent years have seen several strategies developed for inhibiting oncomiRNA in vivo [15]. Hence, in this study, we focused on overexpressed miRNAs that can regulate the intestinal flora in the perioperative period of CRC. The target miRNA adopted in this study was initially selected by analyzing the miRNA database and summarizing the literature. First, we investigated the ranking order of miRNAs associated with intestinal differentiation, structure, and barrier function [16]. Using the miRCancer database (http://mircancer.ecu.edu), we determined that the aforementioned miRNAs were abnormally expressed in CRC. MiR-21-5p, miR-135-5p, and miR-155-5p all regulate the inflammatory process of digestive diseases through the immune system; this is consistent with previous research [17–19]. In addition, alterations in the expression levels of miR-21-5p [20, 21], miR-135-5p [22], and miR-155-5p [23] in serum can be utilized for the early diagnosis of CRC, prediction of treatment outcomes, and prognosis. It has also been shown that the dysregulation of these three miRNAs may lead to drug resistance, and restoring the dysregulated miRNAs could effectively overcome this resistance. In CRC patients with high miR-21-5p [24], miR-135-5p [25], and miR-155-5p [15] expression, overall survival and disease-free survival rates were significantly worse than in patients with low miR-21-5p, miR-135-5p, and miR-155-5p expression. Moreover, Liang et al. found that the co-delivery of functional miR-21 and anticancer drugs by exosomes reverses colorectal cancer resistance, thereby improving the efficacy of cancer therapy [26]. A novel strategy to sensitize CRC to chemotherapy may involve the use of miR-135 antisense nucleotides [15]. Targeted delivery of anti-miR-155 by functionalized mesoporous silica nanoparticles is a promising treatment for colorectal cancer [27]. Therefore, in this study, we evaluated the dynamic changes in the expression levels of a group of circulating miRNAs, including miR-21-5p, miR-135-5p, and miR-155-5p, before and after the perioperative period of CRC.

Bifidobacterium triplex viable capsule is a co-probiotic that has been extensively used in the diagnosis and treatment of inflammatory diseases of the intestinal tract and CRC. In this study, Bifidobacterium triplex viable capsules were administered as a perioperative probiotic to patients with CRC. Our objective was to investigate the changes in the expression levels of serum circulating miR-21-5p, miR-135-5p, and miR-155-5p in patients with CRC who received Bifidobacterium triplex viable capsules perioperatively. This research will provide a foundation for investigating the mechanism of action of Bifidobacterium triplex viable capsules in promoting perioperative recovery in patients with colorectal cancer.

## Materials and methods

### Patient recruitment

A total of 60 primary patients with CRC admitted to the Department of General Surgery at Shanxi Bethune Hospital between June 2020 and December 2020 were selected with a 3-year follow-up. Using the randomization method, they were randomly divided into two groups: 20 cases in the control group and 40 cases in the experimental group. Blood samples were collected from the Department of General Surgery at Shanxi Bethune Hospital for this study. The Ethics Committee of Shanxi Bethune Hospital (YXLL-2019–062) reviewed and approved the study protocol, and all patients provided informed consent by signing a consent form.

Patients were included based on the following criteria: 1. age between 45 and 85 years old; 2. an imaging examination, intraoperative exploration, and gastrointestinal endoscopy confirmed that the tumor was solitary and had no metastasis; 3. standard radical surgeries for CRC were conducted; 4. pathological analysis of postoperative specimens confirmed the presence of malignant tumors; 5. TNM stages were classified as stages I–III. Patients with the following conditions were excluded: 1. preoperative complications such as intestinal perforation or obstruction; 2. detection of distant metastases through preoperative imaging or intraoperative examination; 3. previous history of oncological surgical treatment; 4. history of colorectal surgery; 5. recent use of antibiotics and probiotics; 6. history of infectious diseases; 7. history of diabetes mellitus, hypertension, or coronary heart disease.

### Perioperative treatment and probiotics

Bifidobacterium triple viable capsules, marketed as Bifico, were the probiotics utilized in the study. The capsule contains *Bifidobacterium longum*, *Lactobacillus*, and *Enterococcus*. Each capsule contains 210 mg of pharmaceutical powder and a minimum of 1.0*10^^7^ CFU of live bacteria. The placebo capsules were indistinguishable from the Bifidobacterium triplex viable capsules in terms of taste and texture. The only difference was that the placebo capsules lacked any live microorganisms. Both samples were prepared in capsule form, placed in aluminum foil pouches, and stored at room temperature. Shanghai Sine Pharmaceutical Laboratories Co., Ltd. manufactured the probiotics and placebos used in the study.

All patients were given semi-liquid food 3 days prior to surgery, full liquid food 1 day prior to surgery, oral Folium Sennae 3 days prior to surgery to cleanse the intestinal tract, no food or liquids for 12 h prior to surgery, and compound polyethylene glycol electrolytes to prepare the intestinal tract until the stools were watery.

The same group of physicians performed the surgery and anesthesia. The probiotic, Bifidobacterium triplex viable capsule, was administered orally for 3 days prior to surgery to the experimental group, with four capsules per dose, taken twice daily. The capsules were consumed with a sip of water after surgery and continued for 7 days. The placebo was administered orally to the control group, and all other procedures were identical to the experimental group. Before and after the skin incision, prophylactic antibiotics and nutritional support were routinely administered to prevent infection.

### Collection of serum samples

Fasting blood was collected from patients 3 days before and 7 days after surgery. Approximately 4 mL of whole blood was drawn from the vein at the elbow and placed in a serum collection tube. The tube was then left at room temperature for 1 h before being centrifuged at 4 °C for 10 min at a low speed of 1200 g/min. After centrifugation, the upper layer of serum was transferred to 1.5 mL EP tubes at 1 mL per tube. To remove any remaining cellular debris from the previous low-speed centrifugation, the tubes were centrifuged at a high speed of 12,000g/min at 4 °C for 5 min. The supernatant was then pipetted and dispensed into new 1.5 mL EP tubes at 500 μL per tube. The top cap of each tube was sealed with a membrane and labeled according to the grouping. The tubes were then placed in a refrigerator with a temperature of − 80 °C for storage.

### RNA extraction and miRNA expression detection

Total RNA was extracted using the Total RNA Extractor manufactured by Sangon Biotech (Shanghai). Using a spectrophotometer, the concentration and purity of the extracted RNA were detected. Reverse transcription was performed using the All-in-One™ miRNA qRT-PCR Detection System 2.0 manufactured by Gene Copoeia. RNA was reverse transcribed into complementary DNA (cDNA). The cDNA was used as a template for quantitative experiments on miRNAs in a PCR instrument using the PerfectStart® Green qPCR SuperMix from TransGen Biotech.

### Statistical analysis

Using the 2^−ΔΔCt^ method, the experimental data were processed and analyzed. In this method, △*Ct *_*preoperative*_ = the mean ± standard deviation of (the *Ct* value of the target gene minus the *Ct* value of the internal reference). Similarly, △*Ct *_*postoperative*_ = the mean ± standard deviation of (the *Ct* value of the target gene minus the *Ct* value of the internal reference). Finally, △△*Ct* = the mean ± standard deviation of (the postoperative △*Ct* minus the preoperative △*Ct*).

SPSS 25.0 software was used for statistical analysis, and GraphPad Prism 8 was used to create graphs. The paired *t* test was used to compare the relative expression content of miRNAs in serum within the two groups. Using a *t* test for independent samples, the relative expression content of miRNAs between the two groups was compared. Using a chi-squared test or Fisher’s exact test, a comparison of the counting data was performed.

The follow-up period was defined as the time interval between the date of informed consent until death or last clinical contact (Dec, 2023). Disease-free survival (DFS) is defined as the time from randomization to tumor recurrence of primary cancer, new CRC, distant metastases, or death from any cause, whichever comes first. We compared patient survival using the Kaplan–Meier method and the log-rank test. The significance level of the test was set at 0.05, and a *P* value of ≤ 0.05 indicated that the differences observed were statistically significant.

## Results

### Comparison of general data of patients in the two groups

**Fig. 1:**
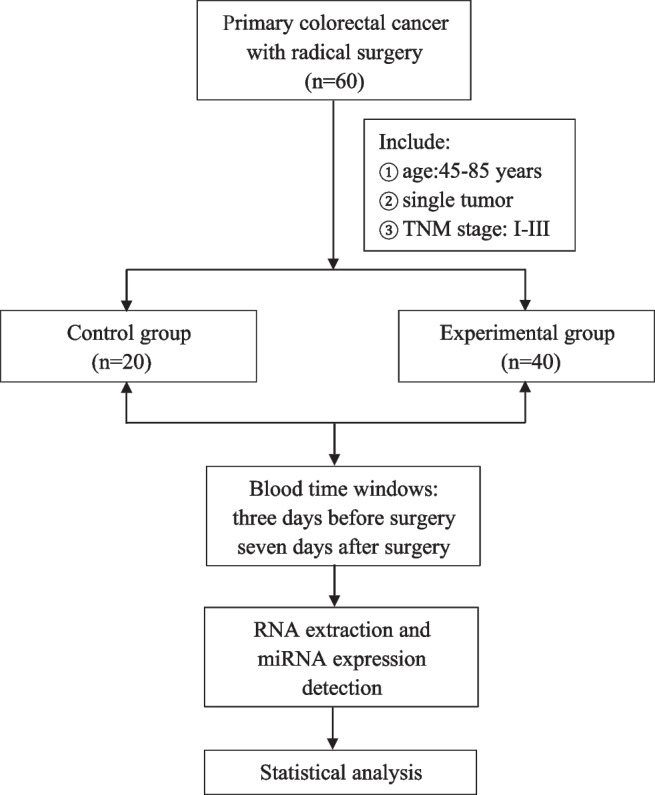
Study design and workflow

This investigation included a total of 60 patients with CRC, with 20 cases in the control group and 40 cases in the experimental group (Fig. 1). The average age of enrollment was 66 years old; 51.7% (31/60) of the participants were male, while 48.3% (29/60) were female. In terms of lesion location, 30% of the tumors were found in the ascending colon, 15% in the descending colon, 31.7% in the sigmoid colon, and 23.3% in the rectum. According to the TNM staging method, stage I and II CRCs accounted for approximately 56.7%, while stage III CRCs accounted for 43.3%.

There was no statistical difference (*P* > 0.05) between the two groups in terms of patient gender (*P* = 0.465), age (*P* = 0.566), lesion location (*P* = 0.194), and TNM staging (*P* = 0.442). The specific conditions of the enrolled patients are presented in Table 1.

**Table 1:** Basic information of enrolled patients

Group	Control group (*n* = 20)	Experimental group (*n* = 40)	*X*^*2*^ value	*P* value
Gender				
Male	9(45.0%)	22(55.0%)	0.534	0.465
Female	11(55.0%)	18(45.0%)		
Age				
≥ 60	8(40.0%)	13(32.5%)	0.330	0.566
< 60	12(60.0%)	27(67.5%)		
Location of lesion				
Ascending colon	4(20.0%)	14(35.0%)	4.715	0.194
Descending colon	3(15.0%)	6(15.0%)		
Sigmoid colon	5(25.0%)	14(35.0%)		
Rectum	8(40.0%)	6(15.0%)		
TNM				
I	1(5.0%)	5(12.5%)	1.786	0.442
II	8(40.0%)	20(50.0%)		
III	11(55.0%)	15(37.5%)		

### Comparison of postoperative complication occurrence between the two groups

Among the experimental group patients, there was one case of postoperative urinary system infection, resulting in a complication rate of 2.5% (1/40). In the control group, there was one case of postoperative pulmonary infection and one case of urinary system infection, resulting in a complication rate of 10.0% (2/20). The difference between the two groups was statistically significant (*P* < 0.05).

### Differences in the relative expression content of miRNAs in serum between the two groups

**Fig. 2:**
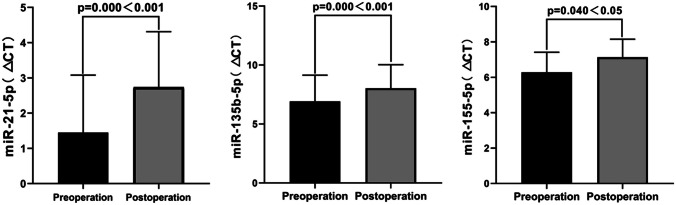
Differences in expression of miR-21-5p, miR-135b-5p, and miR-155-5p in the serum of the control group before and after surgery. △*Ct *_*preoperative*_ = the mean ± standard deviation of (the *Ct* value of the target gene minus the *Ct* value of the internal reference). △*Ct *_*postoperative*_ = the mean ± standard deviation of (the *Ct* value of the target gene minus the *Ct* value of the internal reference)

First, we analyzed and compared the differences in the relative expression levels of miRNAs in the serum of patients between the two groups. In the control group, postoperative expression of miR-21-5p decreased compared to preoperative levels (*t* =  − 5.977, *P* = 0.000). Similarly, miR-135-5p expression also decreased postoperatively compared to preoperative levels (*t* =  − 5.522, *P* = 0.000). In addition, miR-155-5p expression decreased postoperatively compared to preoperative levels (*t* =  − 2.201, *P* = 0.040). Figure 2 illustrates the specific changes in the differences between the preoperative and postoperative periods. All three miRNAs detected in this study showed a decrease in expression after surgery (*P* < 0.05). Among them, the significant difference in expression levels was larger for miR-21-5p and miR-135-5p (*P* < 0.001).

**Fig. 3:**
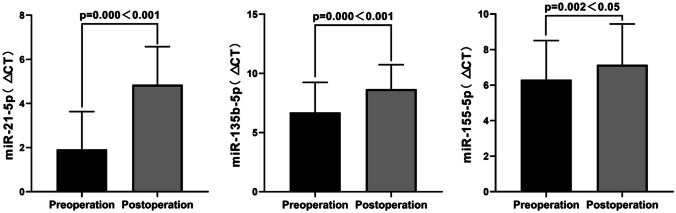
Differences in expression of miR-21-5p, miR-135b-5p, and miR-155-5p in the serum of the experimental group before and after surgery. △*Ct *_*preoperative*_ = the mean ± standard deviation of (the *Ct* value of the target gene minus the *Ct* value of the internal reference). △*Ct *_*postoperative*_ = the mean ± standard deviation of (the *Ct* value of the target gene minus the *Ct* value of the internal reference)

Compared to the preoperative period, the expression of miR-21-5p decreased significantly in the experimental group patients (*t* =  − 11.361, *P* = 0.000). Similarly, the expression of miR-135-5p also decreased compared to the preoperative period (*t* =  − 9.006, *P* = 0.000), as did the expression of miR-155-5p (*t* =  − 3.377, *P* = 0.002). Figure 3 illustrates the specific changes in the difference between the preoperative and postoperative periods. Before and after the surgery, the three previously mentioned miRNAs exhibited the same trend of change in the experimental group as observed in the control group.

### Differences in the relative expression levels of miRNAs in serum between groups

**Fig. 4:**
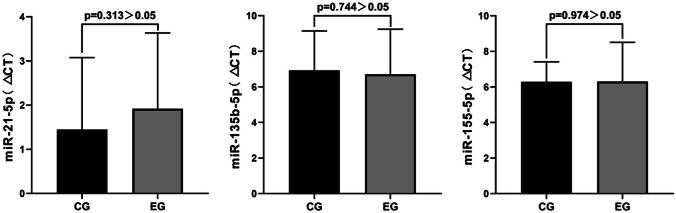
Differences in expression of miR-21-5p, miR-135b-5p, and miR-155-5p in the serum between two groups of patients before intervention with placebo or Bifidobacterium triple viable capsules. △*Ct *_*preoperative*_ = the mean ± standard deviation of (the *Ct* value of the target gene minus the *Ct* value of the internal reference). △*Ct *_*postoperative*_ = the mean ± standard deviation of (the *Ct* value of the target gene minus the *Ct* value of the internal reference)

In addition, we compared and analyzed the differences in the relative expression levels of miRNAs in serum between the control group and the experimental group before and after the intervention of placebo or Bifidobacterium triplex viable capsules. Figure 4 demonstrates that there was no significant difference in the expression of miR-21-5p (*P* = 0.313), miR-135-5p (*P* = 0.744), and miR-155-5p (*P* = 0.974) between the two groups of patients before the intervention of placebo or Bifidobacterium triplex viable capsule (*P* > 0.05).

**Fig. 5:**
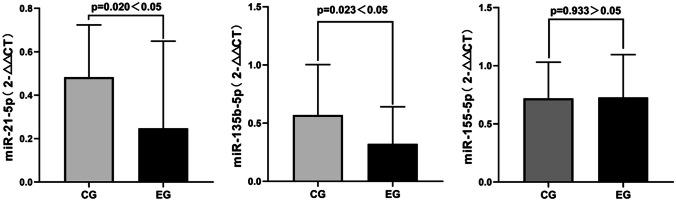
Differences in expression of miR-21-5p, miR-135b-5p, and miR-155-5p in the serum between two groups of patients after intervention with placebo or Bifidobacterium triple viable capsules. △*Ct *_*preoperative*_ = the mean ± standard deviation of (the *Ct* value of the target gene minus the *Ct* value of the internal reference). △*Ct *_*postoperative*_ = the mean ± standard deviation of (the *Ct* value of the target gene minus the *Ct* value of the internal reference). △△*Ct* = the mean ± standard deviation of (the postoperative △*Ct* minus the preoperative △*Ct*)

The expression of miR-21-5p (*t* =  − 2.398, *P* = 0.020) and miR-135-5p (*t* =  − 2.343, *P* = 0.023) decreased in the experimental group compared to the control group after the administration of probiotics (7 days postoperatively). However, there was no significant change in the relative expression of miR-155-5p (*t* = 0.084, *P* = 0.933). These findings suggest that probiotics have a certain impact on the expression of miRNAs, as depicted in Fig. 5.

## Relationship between the levels of miRNAs after surgery and patients’ survival

**Fig. 6:**
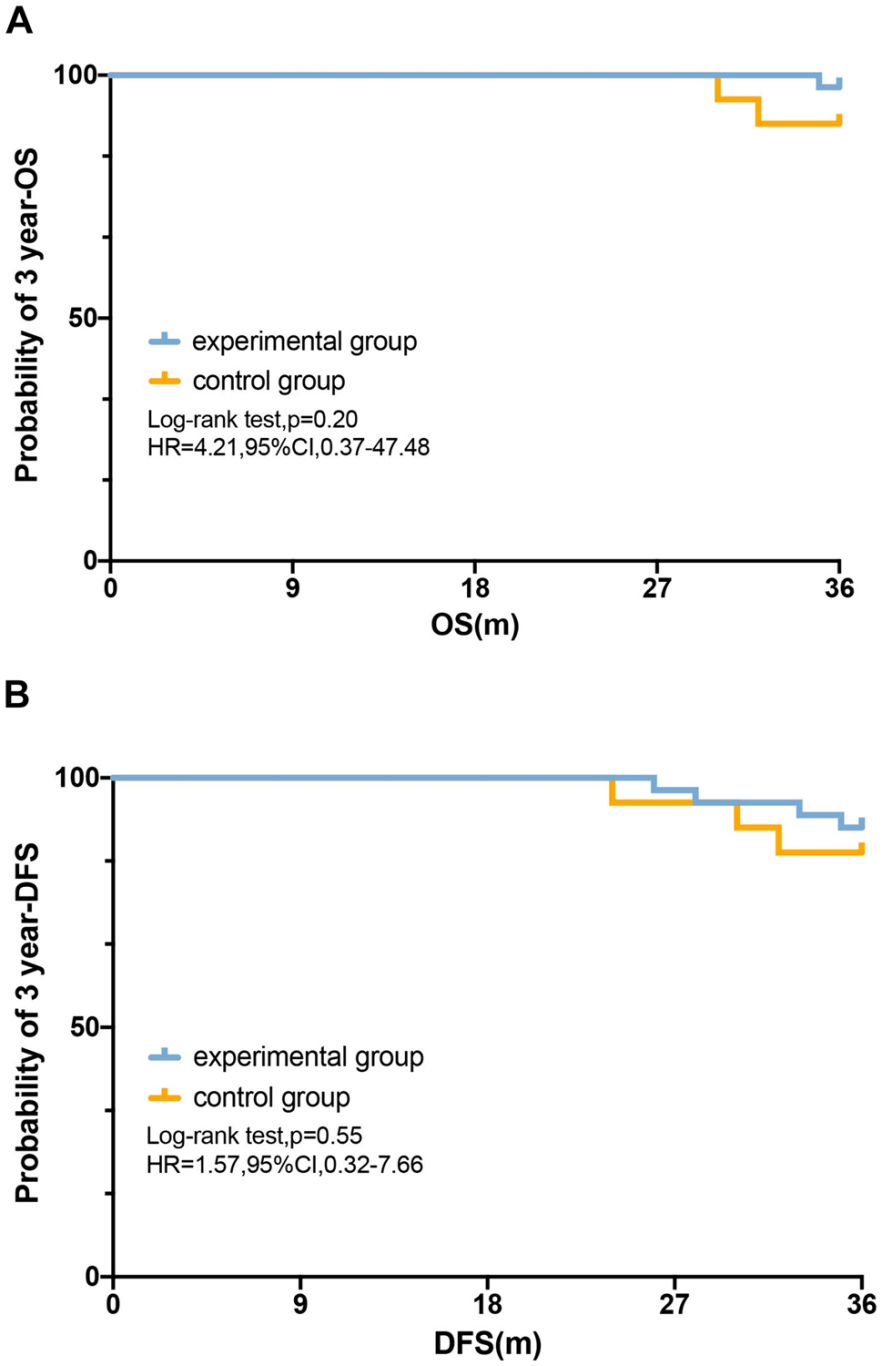
Relationship between the levels of miRNAs after surgery and patients’ survival. Kaplan–Meier curves provided estimates of 3-year OS (**A**) and DFS (**B**) as determined by the levels of miRNAs after surgery

Finally, we compared and analyzed the differences in the OS and DFS between the two groups. In the Kaplan–Meier survival curve analysis of all patients, 3-year OS (HR = 4.21; 95% CI 0.37–47.48; log-rank *P* = 0.20) or 3-year DFS (HR = 1.57; 95% CI 0.32–7.66; log-rank *P* = 0.55) rates of the experimental patients with lower miR-21-5p, miR-135-5p, and miR-155-5p expression were not significantly longer than these patients in the control group (Fig. 6).

## Discussion

In studies investigating the relationship between CRC and intestinal flora, researchers discovered that the diversity and abundance of bacteria in the intestinal mucosa and feces of patients with CRC are significantly altered. Specifically, the levels of beneficial bacteria such as *Bifidobacterium* and *Faecalibacterium* tend to decrease, while the levels of harmful bacteria such as *E. faecalis* and *Fusobacterium nucleatum* tend to increase [11]. This imbalance in intestinal bacterial composition results in varying degrees of intestinal flora dysbiosis, which in turn increases permeability of the intestinal mucosa and levels of inflammatory factors. It is believed that these changes play a crucial role in the development and progression of CRC [28]. Probiotic supplementation can effectively regulate the composition of the intestinal flora and influence the development and treatment of CRC [12, 29, 30]. However, its specific mechanism is still unclear.

In this investigation, we conducted a study on patients with CRC who received perioperative Bifidobacterium triplex viable capsules. We found that the incidence of postoperative complications was lower in the experimental group than in the control group (2.5% vs. 10.0%). Using the expression of circulating miRNAs as the primary observational index, the effect of using Bifidobacterium triplex viable capsules on the expression of serum miRNAs in patients with CRC was investigated. The results indicated that both experimental and control group patients showed a decrease in the expression content of miR-21-5p, miR-135-5p, and miR-155-5p 7 days after surgery (*P* < 0.05). Among them, miR-21-5p and miR-135-5p were significantly different (*P* < 0.001), indicating that radical surgery to remove the tumor load can affect the expression of oncogenic miRNA content in serum. This result is consistent with previous research findings. Both Toiyam et al. [20] and Li et al. [21] analyzed serum miR-21-5p levels comparatively between patients with CRC and healthy populations, and the results showed that serum miR-21-5p levels were higher in patients with CRC than in healthy populations preoperatively and that miR-21-5p levels were significantly lower in the postoperative period compared to the preoperative period (*P* < 0.001). The decrease in serum miR-21-5p (*P* = 0.020) and miR-135-5p (*P* = 0.023) expression after the intervention of Bifidobacterium triplex capsules was obvious, suggesting that Bifidobacterium triplex viable capsules could reduce the expression of oncogenic miRNAs in the serum; meanwhile, the trend of the above-mentioned miRNA expression content suggests that the use of Bifidobacterium triplex viable capsules may be beneficial to the treatment of tumors. Heydari et al. [31] also discovered that *Lactobacillus* and *Bifidobacterium* enhanced the therapeutic efficacy of colon cancer treatment in a mouse model of colon cancer by increasing the expression of oncogenic miRNAs and their target genes and decreasing the expression of oncogenic genes. Regarding its specific mechanism, miR-21-5p acts as a pro-oncogenic factor. MiR-21-5p may be activated by the phosphatidylinositol-3-kinase (PI3K), signal transducer and activator of transcription 3 (STAT3), and programmed cell death factor 4/tumor necrosis factor-α (PDCD4/TNF-α) signaling pathways, which affect inflammatory processes and tumor progression in CRC [32]. Meanwhile, they play a role in regulating the inflammatory process of CRC, which may synergize with intestinal flora dysbiosis to regulate downstream inflammatory factors (e.g., interleukin-6, TNF-α) and control the inflammatory process of tumors. In addition, probiotics can alter the structure of the intestinal flora and increase the population of beneficial bacteria. This can help combat the persistent inflammatory response caused by dysbiosis and potentially have a therapeutic effect on CRC by regulating tumor-associated pathways [12]. miR-135-5p is a cancer-promoting factor, and the high expression of miR-135-5p in patients with CRC is associated with adenomatous polyposis coli (APC) gene deletion, phosphatase, and tensin homolog (PTEN)/PI3K pathway dysregulation, and altered expression levels of circulating RNA circNOL10 [33, 34]. In the study by Han et al., interleukin 1 upregulated miR-135b to promote inflammation-associated gastric carcinogenesis in mice, suggesting that miR-135-5p promotes the inflammatory process in digestive diseases. In contrast, miR-155-5p promotes the progression of CRC by targeting the negative regulator suppressor of cytokine signaling 1 (SOCS1) and SH2 domain-containing inositol 5-phosphatase 1 (SHIP1) to enhance Toll-like receptor 4 polypeptide (TLR4) signaling; TLR4 activation induces miR-155-5p expression through transcriptional and post-transcriptional mechanisms [35]. The expression level of miR-155-5p in the experimental group decreased significantly after surgery compared with that in the control group before and after surgery (*P* = 0.002). However, compared with miR-21-5p and miR-135-5p, there was no statistical difference before and after surgery in miR-155-5p (*P* = 0.933), which may be due to the deviation of the limited sample size in this study. This may be one of the mechanisms by which the intestinal flora improves the expression level of miRNAs; however, further research is required to confirm this hypothesis.

Traditionally, the TNM stage has been the main classification system guiding prognosis. However, in the recent decade, greater emphasis on molecular biomarkers such as microsatellite instability (MSI) [36], kirsten rat sarcoma virus (KRAS) [36], circulating tumor DNA (ctDNA) [37], and miRNAs [38], improves the accuracy of prognostication. In this present study, there was no significant statistical difference of the 3-year OS (HR = 4.21; 95% CI 0.37–47.48; log-rank *P* = 0.20) or 3-year DFS (HR = 1.57; 95% CI 0.32–7.66; log-rank *P* = 0.55) in two groups. However, the miR-21-5p [24], miR-135-5p [25], and miR-155-5p [15] expression levels in CRC patients were independent prognostic factors for OS and DFS. Possibly, this is due to our short follow-up time or the stage I–III in the study population or the fact that our study only included perioperative oral probiotics.

The primary limitation of this investigation is the small sample size, and the results of the study must be confirmed by a larger sample size. To further investigate the mechanism of action of Bifidobacterium triplex viable capsules in promoting the perioperative recovery of patients with CRC, we established an oral placebo group as the control group, but healthy people were not included. This was done to exclude any potential effects on intestinal flora caused by the prophylactic use of antibiotics and bowel preparation that were clinically necessary. This may differ slightly from the real world. It is worth noting that in this study, only quantitative reverse transcription polymerase chain reaction (qRT-PCR) was used, which is the gold standard tool to detect miRNAs, and the results of the study were preliminary, and in further studies, RNA sequencing will be used simultaneously to support our research, and all enrolled patients’ survival outcomes will be collected. Studying survival data from experimental and control groups and comparing whether there is a statistical difference, multivariate analysis was not used to confirm the association between the patients’ stratification, clinicopathologic data, and circulating miRNA expression, to test the hypothesis that Bifidobacterium triplex viable capsules might downregulate the expression level of oncomiRNA to enhance the CRC patients’ anti-cancer ability, providing a potential for anti-cancer treatment. In addition, the mechanism of targeted miRNAs affecting patients’ disease course and the correlation between the colonization of probiotics in patients and miRNA changes also need to be further analyzed.

In conclusion, the utilization of Bifidobacterium triplex viable capsules has the potential to enhance the postoperative recuperation of patients with CRC. This effect is achieved through significant modulation of the expression of miR-21-5p and miR-135-5p in the serum. These modifications in miRNA expression may be the reason why Bifidobacterium triplex viable capsules promote perioperative recovery in patients with colorectal cancer. This research seeks to contribute additional evidence supporting the optimal utilization of probiotic-based therapeutics in clinical settings.

## Data Availability

All data generated or analyzed during this study are included in this article. Further enquiries can be directed to the corresponding author.
